# Effects of an eight-week French contrast training program on lower-limb explosive power, acceleration, and muscle strength in male college badminton players

**DOI:** 10.3389/fphys.2026.1777883

**Published:** 2026-04-02

**Authors:** Kaixiang Zhou, Ruting Lin, Ziren Zhao, Na Yu, Xin Zheng, Jinhong Li, Nijiao Deng, Yu’an Sun

**Affiliations:** 1 College of Physical Education and Health Science, Chongqing Normal University, Chongqing, China; 2 College of Education, Beijing Sport University, Beijing, China; 3 College of Sports and Health, Chengdu University of Traditional Chinese Medicine, Chengdu, Sichuan, China

**Keywords:** acceleration, French contrast training, maximal strength, mean concentric velocity, sprint, vertical jump

## Abstract

**Background:**

Badminton is a high-intensity sport that demands lower-limb athletic performance. French Contrast Training (FCT), which combines heavy compound exercises, plyometrics, light to moderate loads, and assisted plyometric movements in one session, has been suggested to enhance neuromuscular adaptations more effectively. However, the efficacy of FCT on lower-limb explosive power, acceleration, and muscle strength compared to equal-load training (ELT) in badminton players remains unclear.

**Objective:**

This study aims to investigate the effects of an eight-week FCT program on lower-limb explosive power, acceleration, and muscle strength compared to ELT in college badminton players.

**Methods:**

Thirty male college badminton players were randomly assigned to the FCT group (n=15) or the ELT group (n=15). Both groups completed an eight-week (twice a week) training program. The FCT protocol consisted of four sequential exercises per session: 80% 1RM back squat, 30cm hurdle jump, 30% 1RM barbell squat jump, and band-assisted jump. The control group performed ELT, an isolated method that involved exercises with loads aligned to FCT. Outcomes measurement before and after training included countermovement jump (CMJ), squat jump (SJ), 10-meter sprint time, maximal velocity (V_max_), maximal acceleration (A_max_), 1RM back squat, and mean concentric velocity (MCV) from 20% to 80%1RM. We used two-way repeated measures analysis of variance (ANOVA) (time × group) with Bonferroni *post hoc* tests, and effect sizes were reported as partial eta squared ( $\eta_{p}^{2}$). p< 0.05 was statistically significant.

**Results:**

FCT yielded greater improvements than ELT in CMJ (p< 0.001, $\eta_{p}^{2}$ = 0.621), SJ (p< 0.001, $\eta_{p}^{2}$ = 0.849), 10-meter sprint time (p< 0.001, $\eta_{p}^{2}$ = 0.853), V_max_ (p< 0.001, $\eta_{p}^{2}$ = 0.638), A_max_ (p< 0.001, $\eta_{p}^{2}$ = 0.820), and MCV from 20 to 40% 1RM (p< 0.001, $\eta_{p}^{2}$ = 0.615; p< 0.001, $\eta_{p}^{2}$ = 0.697). However, no significant differences were found between the groups for the 1RM back squat (p = 0.218, $\eta_{p}^{2}$ = 0.054), and MCV from 60 to 80%1RM (p = 0.579, $\eta_{p}^{2}$ = 0.011; p = 0.900, $\eta_{p}^{2}$ = 0.001).

**Conclusion:**

This study suggests that French Contrast Training is an effective strategy for improving lower-limb explosive power and rapid acceleration in male college badminton players, but it does not further improve muscle strength.

## 1 Introduction

Badminton is a high-intensity, intermittent racket sport that requires players to perform repeated bouts of explosive movements such as rapid accelerations, multidirectional sprints, jumps, and powerful smashes within short recovery intervals (Phomsoupha and Laffaye, 2015; Edel et al., 2023; Rusdiana et al., 2023). Success in competitive badminton depends mainly on the athlete’s ability to generate force quickly, change direction efficiently, and sustain high-intensity actions throughout extended rallies (Wang et al., 2025). Accordingly, lower-limb explosive power, acceleration ability, and maximal strength are recognized as fundamental physical qualities underpinning the athletic performance of badminton players (Maloney, 2018; Fernandez-Fernandez et al., 2022; Hu et al., 2025). Typically, resistance training is a common method for enhancing lower-limb strength and explosive power in badminton players (Comfort et al., 2023; Lu et al., 2025). For example, Wang et al. showed that resistance training increased strength and power for badminton athletes (Wang et al., 2025). However, this method employs isolated training strategies that provide limited stimulus across the full force-velocity (F-V) curve (Issurin, 2008; Wang et al., 2023; Riscart-López et al., 2025). For example, Cormie et al. confirmed that resistance training predominantly targets the high-force, low-velocity region of the F-V curve. It provides limited stimulus to the high-velocity region, which constitutes a methodological limitation for developing rapid production and stretch-shortening cycle (SSC) efficiency (Cormie et al., 2011a). Studies suggested that this method has limited stimulating effects on the F-V curve, potentially restricting athletes’ ability to generate rapid force (Cormie et al., 2011b; Issurin, 2016; Pagaduan and Pojskic, 2020). Samozino et al. reported that training targeting only one side of the F-V curve, which may lead to long-term imbalance, can limit the development of explosive, rapid force production skills (Samozino et al., 2014). Therefore, stimulating the full F-V curve is crucial for enhancing explosive and rapid force production.

French Contrast Training (FCT) has gained attention for its potential to enhance athletic performance by integrating heavy compound exercises, plyometric exercises, light-to-moderate load exercises, and assisted plyometric exercises in a single session, thereby stimulating adaptations across the full F-V curve (Noufal et al., 2024). Specifically, FCT combines four phases within a single session: 1) heavy compound exercises (e.g., 80 to 90%1RM back squat); 2) plyometric exercises (e.g., vertical jumps); 3) light-to-moderate load exercises (e.g., 25 to 45% 1RM barbell weighted jumps); and 4) assisted plyometric exercises (e.g., band-assisted jumps) (Hernández-Preciado et al., 2018; Salam and Sherif, 2020; Chen et al., 2025). Through these complementary stimuli across the full F-V curve to enhance neuromuscular function. Additionally, following post-activation performance enhancement (PAPE) principles, FCT may activate the central nervous system and improve force output (Cengizel and Şenel, 2025; Karabel and Makaracı, 2025). For example, Welch et al. reported that a 6-week FCT program improved vertical jump height in weight-trained male players (Welch et al., 2019). Valappil et al. reported that a 12-week FCT program enhances sprint times for male hockey players (Valappil et al., 2024), consistent with our previous systematic review and meta-analysis, which demonstrated that FCT is an effective method for improving vertical jump height and sprint performance (Zhao et al., 2025). Although FCT has shown potential to enhance lower-limb athletic performance, there is still no empirical evidence confirming its effectiveness for badminton players. Prior studies on FCT mainly compare it with resistance training, complex training, or routine tactic training. However, the improvements in lower-limb athletic performance may be attributed either to the unique structure of FCT or simply to a higher overall training volume.

This study aims to address this gap by evaluating the effects of an eight-week FCT on lower-limb athletic performance compared to equal-load training (ELT) in college badminton players. To assess these effects, we measured countermovement jump (CMJ), squat jump (SJ), 10-metre sprint time, maximal velocity (Vmax), maximal acceleration (Amax), 1RM back squat and mean concentric velocity (MCV) at 20-80% 1RM. We hypothesized that FCT would produce significantly greater improvements in CMJ, SJ, sprint time, Vmax, Amax, 1RM back squat, and MCV at 20-80% 1RM.

## 2 Materials and methods

### 2.1 Participants

Sample size estimation was informed by a previous study conducted by Rebelo et al, which found a between-group effect size of 0.81 for CMJ (Rebelo et al., 2023). We calculated, using G*Power (ANOVA, f = 0.40, α = 0.05, power = 0.95), that this study requires 24 participants. We recruited thirty badminton players and randomly allocated to the FCT group (n = 15) or the ELT group (n = 15). All participants were members of the university badminton team with at least six years of training experience; they engaged in specialized badminton technique training three times per week; and had no history of lower limb injuries within the past three months. The study was approved by the Ethics Committee of the College of Physical Education and Health Science, Chongqing Normal University (CNU-PSY-202509-006), and all procedures were conducted by the Declaration of Helsinki. Before the experiment, participants were informed of the benefits and potential risks related to the study, and all signed the informed consent form.

### 2.2 Study design

**Figure 1:**
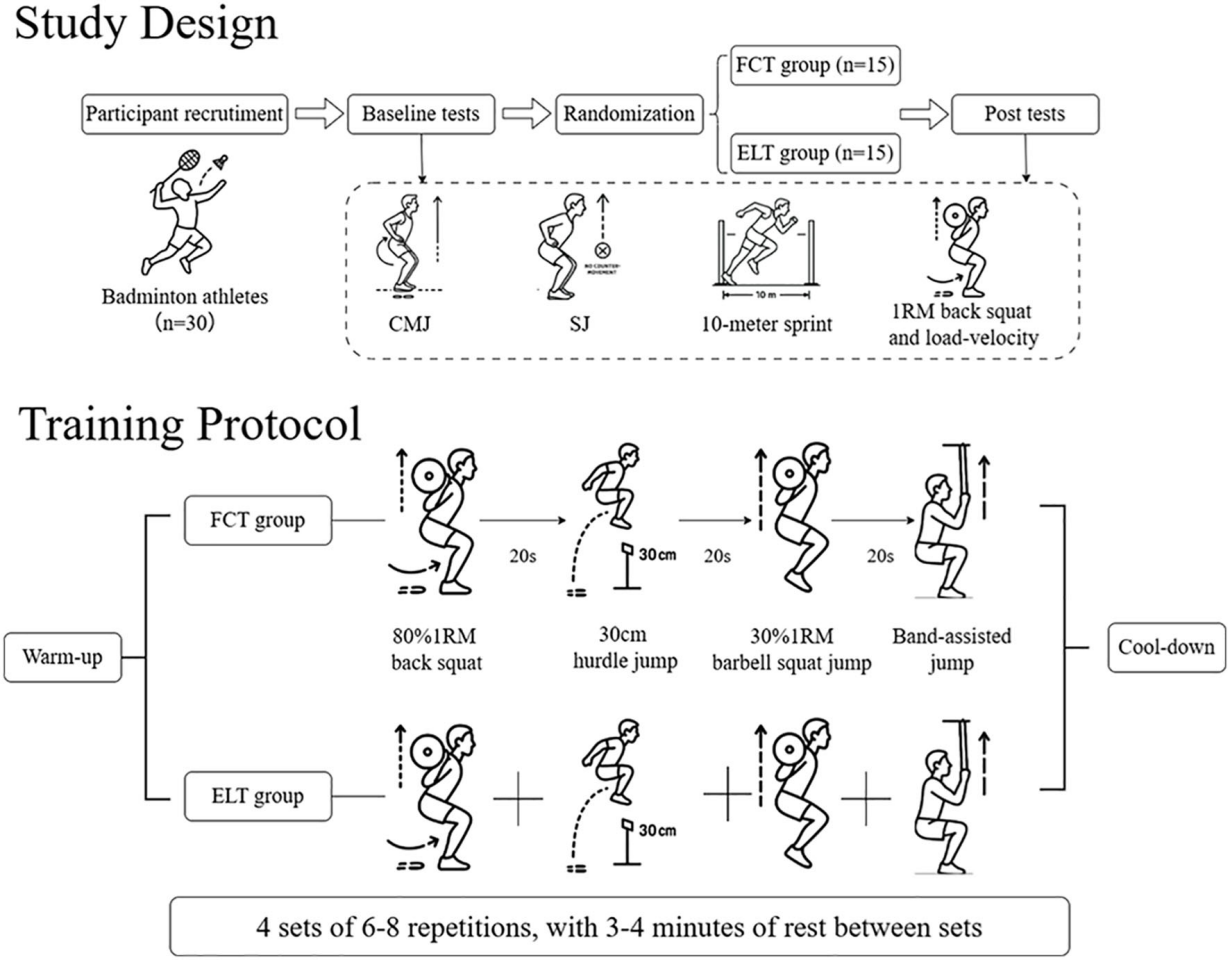
Study design and training protocol.

This study adopted an eight-week, single-blind (assessor-blind) randomized controlled trial design (**Figure 1**). Participants were randomly assigned to either the FCT group or the ELT group using a computer-generated randomization sequence. The intervention was conducted during the preparatory phase of the competitive season. Before baseline testing, all participants completed a familiarization session to standardize training procedures and testing protocols. Both groups trained twice weekly, with at least 48 hours of rest between sessions. Certified strength and conditioning coaches supervised standardized instructions to ensure consistent training implementation and evaluation. To control potential circadian rhythm effects, all testing sessions were conducted at the same time of day for each participant. Participants were instructed to refrain from strenuous physical activity for 48 hours before each testing session and to avoid consuming alcohol and caffeine within 24 hours of testing.

#### 2.2.1 Training protocol

Participants in the FCT group performed a structured, four-phase sequence. Each sequence comprises: 1) heavy compound exercise (80% 1RM squat); 2) plyometric exercise (30cm hurdle jumps); 3) light-to-moderate load exercise (25% 1RM squat jumps); 4) assisted plyometric exercise (band-assisted jumps). Participants completed four sets of each exercise, with 6–8 repetitions per set, rest for 20 seconds within sets, and rest for 3–4 minutes between sets. The 20-second rest period within sets was aligned with previous FCT studies and FCT guidelines (Long et al., 2023; Türkarslan and Deliceoglu, 2024). Participants in the ELT group performed the same exercises, loads, sets, and repetitions as in FCT, but in a traditional blocked format (all sets of one exercise completed before moving to the next). All participants began with a standardized 10-minute warm-up and concluded with a 5-minute cool-down (**Figure 1**). All participants practiced badminton techniques with identical content and duration three times a week during the experiment.

#### 2.2.2 Outcomes measurement

##### 2.2.2.1 Countermovement jump test

We evaluated lower-limb explosive power with CMJ. Participants stood on a contact platform kit (Chronojump Boscosystem, ≥1000 Hz, Spain) with both hands on the iliac crests to minimize arm swing. After an auditory signal, participants rapidly descended into a squat position and immediately performed a vertical jump (Van Hooren and Zolotarjova, 2017). Each participant completed three maximal attempts with 1-minute rest periods, and the best jump height (cm) was recorded for analysis.

##### 2.2.2.2 Squat jump test

We assessed lower-limb concentric explosive performance with the SJ, which minimizes the influence of the stretch-shortening cycle. Participants began in a static squat position on a contact platform kit (Chronojump Boscosystem, ≥1000 Hz, Spain) with their knees flexed at 90°, as verified by a handheld goniometer, while maintaining their hands fixed on the iliac crests to minimize arm swing. After an auditory cue, participants executed a maximal vertical jump (Van Hooren and Zolotarjova, 2017). Each participant completed three maximal attempts with 1-minute rests, and the best jump height (cm) was recorded for analysis.

##### 2.2.2.3 10-meter sprint test

We used the 10-meter sprint test to assess initial acceleration and recorded sprint kinematics using a pull-cord linear encoder (Race Analyzer Kit, Chronojump Boscosystem, Spain). The Race Analyzer Kit has been shown to reliably measure speed (Carmona et al., 2025). Following the manufacturer’s Race Analyzer tutorial, participants completed a standardized warm-up and then stood in a split stance, starting 0.5m behind the line. After an auditory cue, participants sprinted maximally through 15m to avoid early deceleration. Each participant completed three maximal trials, with 2–3 minutes of rest between trials. The best trial times (s), Vmax (maximal velocity, m/s), and Amax (maximal instantaneous acceleration, m/s²) were recorded for analysis.

##### 2.2.2.4 Back squat test

We used the 1RM back squat test to assess maximal dynamic lower-limb strength. We used red elastic bands on the barbell rack to mark each participant’s squat depth (the thigh was parallel to the floor). Participants performed a controlled eccentric phase (~3 seconds), then immediately executed a maximal-effort concentric phase (Keiner et al., 2025). The 1RM back squat test procedure (Shen et al., 2025) was as follows: 1) 10 repetitions at 20kg; 2) 5 repetitions at 50% of the estimated 1RM; 3) 3 repetitions at 75% of the estimated 1RM, and 4) 1 repetition at 90% of the estimated 1RM. We then increased the load by 5-10kg increments until a failed attempt occurred and determined the final 1RM within six attempts.

After establishing 1RM, participants rested for 10 minutes and performed 2–3 repetitions at 20%, 40%, 60%, and 80% of their individual 1RM with maximal concentric intent. We recorded barbell MCV velocity (MCV, m/s) using a linear encoder kit (Chronojump Boscosystem, ≥1000 Hz, Barcelona, Spain) to evaluate the load-velocity relationship. We followed the manufacturer’s Encoder tutorial and user manual. The encoder was fixed to the rack, the tether was clipped to the barbell, and the slack was removed before each set. We sampled the data using the manufacturer’s software, providing 2–3 minutes of rest between each attempt. Trials with depth violations, technical faults, or measurement artifacts were repeated.

### 2.3 Statistical analysis

We presented all data as the mean ± standard deviation (SD) and checked for normality using the Shapiro–Wilk test. We conducted an analysis of outcomes using a two-way (time × group) repeated measures ANOVA, with *post hoc* analyses performed using the Bonferroni test. We expressed effect size as partial eta-squared ( $\eta_{\text{p}}^{2}$), where effect sizes were categorized as trivial ( $\eta_{\text{p}}^{2}$<0.01), small (0.01≤ $\eta_{\text{p}}^{2}$<0.06), moderate (0.06≤ $\eta_{\text{p}}^{2}$<0.14), and large ( $\eta_{\text{p}}^{2}$≥0.14) effects. We evaluated test-retest reliability using the intraclass correlation coefficient (ICC), with results in **Supplementary Table S1**. Descriptive statistics were now provided only for the percentage change (Δ%). p< 0.05 was considered statistically significant. All analyses were performed using the SPSS statistical package (version 27.0, IBM Statistics, Chicago, IL).

## 3 Results

Baseline characteristics of participants showed no significant difference (p > 0.05) (**Table 1**).

**Table 1:** Baseline participant characteristics by groups.

Characteristics	FCT (n=15)	ELT (n=15)	*P*
CMJ (cm)	44.62 ± 5.12	42.75 ± 5.20	*0.330*
SJ (cm)	42.47 ± 4.22	40.40 ± 7.10	*0.342*
10-m sprint time (s)	1.94 ± 0.05	1.93 ± 0.05	*0.757*
10-m Vmax (m/s)	7.49 ± 0.25	7.26 ± 0.38	*0.061*
10-m Amax (m/s^2^)	7.26 ± 0.53	7.12 ± 0.47	*0.465*
1RM squat (kg)	141.00 ± 20.44	135.33 ± 19.11	*0.439*
MCV 20% 1RM (m/s)	0.91 ± 0.16	0.85 ± 0.13	*0.326*
MCV 40% 1RM (m/s)	0.73 ± 0.13	0.74 ± 0.10	*0.922*
MCV 60% 1RM (m/s)	0.59 ± 0.08	0.61 ± 0.08	*0.513*
MCV 80% 1RM (m/s)	0.46 ± 0.05	0.47 ± 0.07	*0.646*

CMJ, countermovement jump; MCV, mean concentric velocity; SJ, squat jump; 1RM, one repetition maximal.

### 3.1 Vertical jump

#### 3.1.1 CMJ

**Figure 2:**
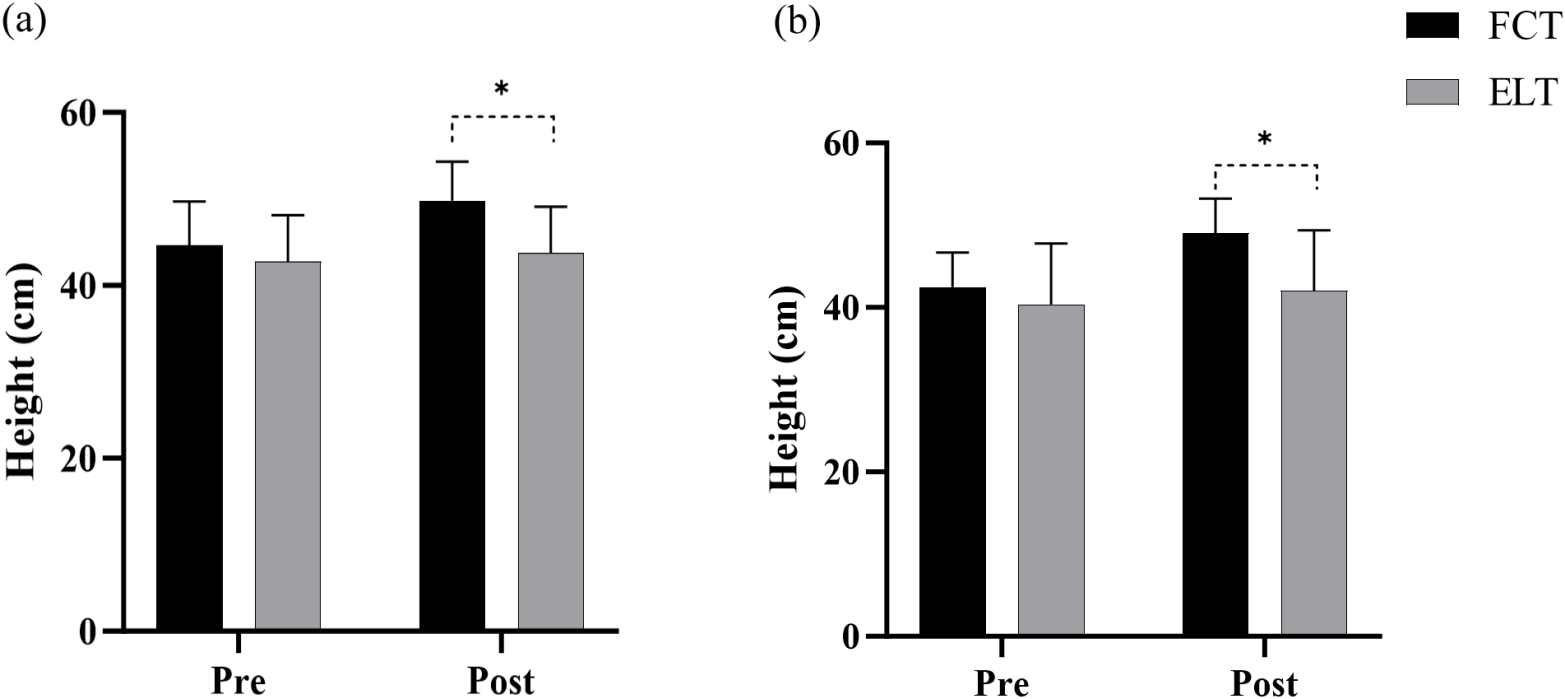
Effect of FCT on the vertical jump. **(a)** CMJ; **(b)** SJ; *p<0.05.

A significant interaction effect between time and group was observed (F = 45.93, p< 0.001, $\eta_{\text{p}}^{2}$ = 0.621, large effect). The main effect of time showed a significant difference (F = 97.71, p< 0.001, $\eta_{\text{p}}^{2}\;$= 0.777, large effect). The main effect of the group showed a significant difference (F = 4.90, p = 0.035, $\eta_{\text{p}}^{2}$ = 0.149, large effect) (**Figure 2**).

**Figure 3:**
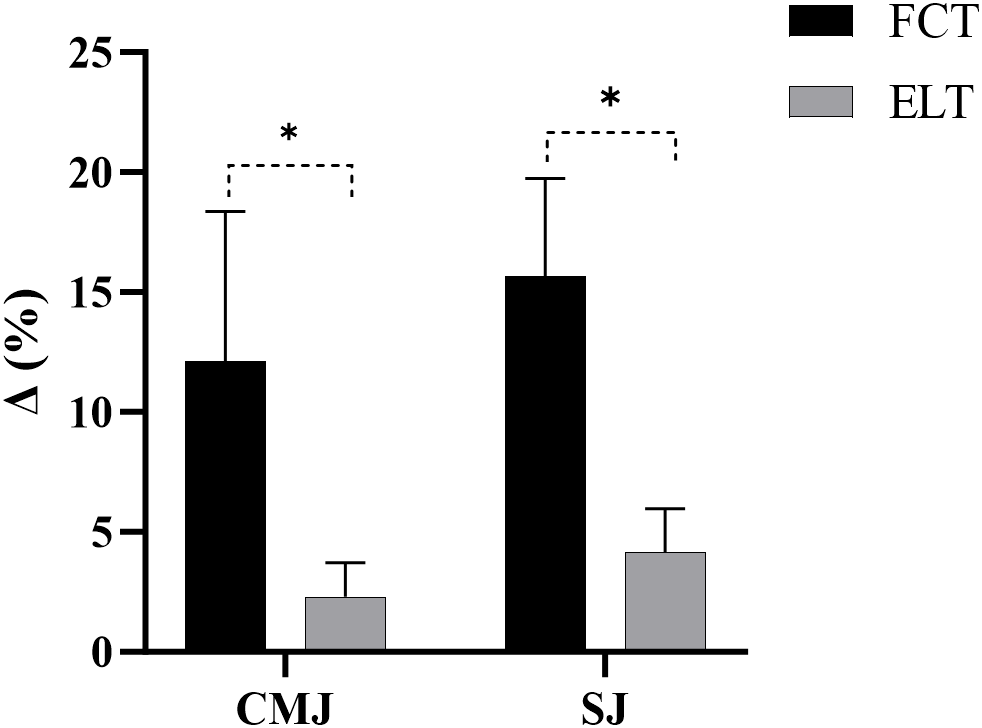
The growth rate of vertical jump. CMJ, countermovement jump; SJ, squat jump; *p<0.05; Δ, growth rate.

The FCT group increased by 12.11% (from 44.62 to 49.84cm), compared to 2.31% (from 42.75 to 43.72cm) in the ELT group (**Figure 3**).

#### 3.1.2 SJ

A significant interaction effect between time and group was observed (F = 157.69, p< 0.001, $\eta_{\text{p}}^{2}$ = 0.849, large effect). The main effect of time showed a significant difference (F = 433.867, p< 0.001, $\eta_{\text{p}}^{2}$ = 0.939, large effect). The main effect of the group showed a significant difference (F = 4.58, p = 0.041, $\eta_{\text{p}}^{2}$ = 0.140, large effect) (**Figure 2**).

The FCT group increased by 15.68% (from 42.47 to 49.04cm), compared to 4.16% (from 40.40 to 42.03cm) in the ELT group (**Figure 3**).

### 3.2 10-meter sprint

#### 3.2.1 Time

**Figure 4:**
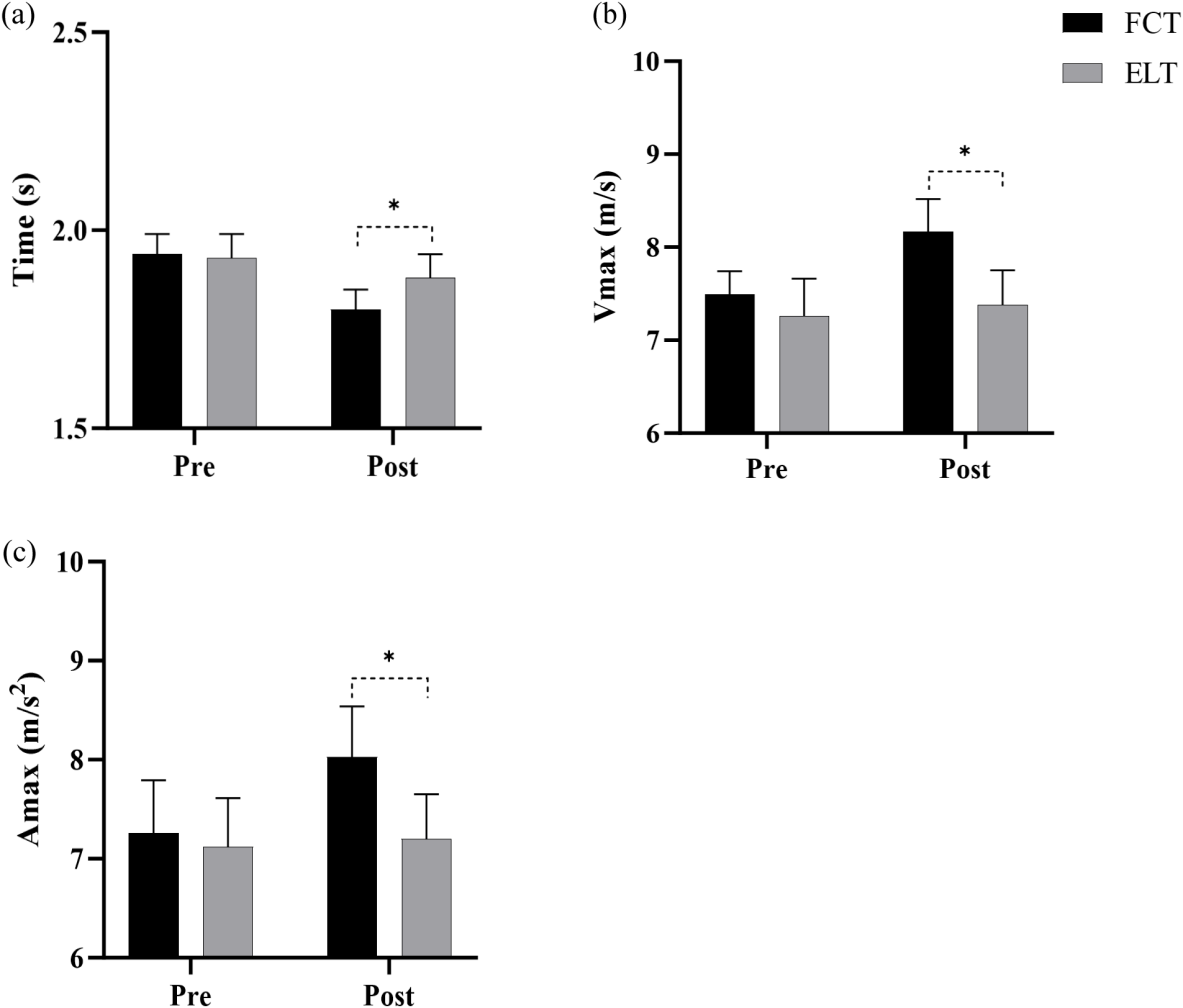
Effect of FCT on the 10-meter sprint. **(a)** Time, **(b)** Vmax, **(c)** Amax. Amax, maximal acceleration; Vmax, maximal velocity; *p<0.05.

A significant interaction effect between time and group was observed (F = 161.95, p< 0.001, $\eta_{\text{p}}^{2}$ = 0.853, large effect). The main effect of time showed a significant difference (F = 646.83, p< 0.001, $\eta_{\text{p}}^{2}$ = 0.959, large effect). The main effect of the group showed a significant difference (F = 5.07, p = 0.032, $\eta_{\text{p}}^{2}$ = 0.153, large effect) (**Figure 4**).

**Figure 5:**
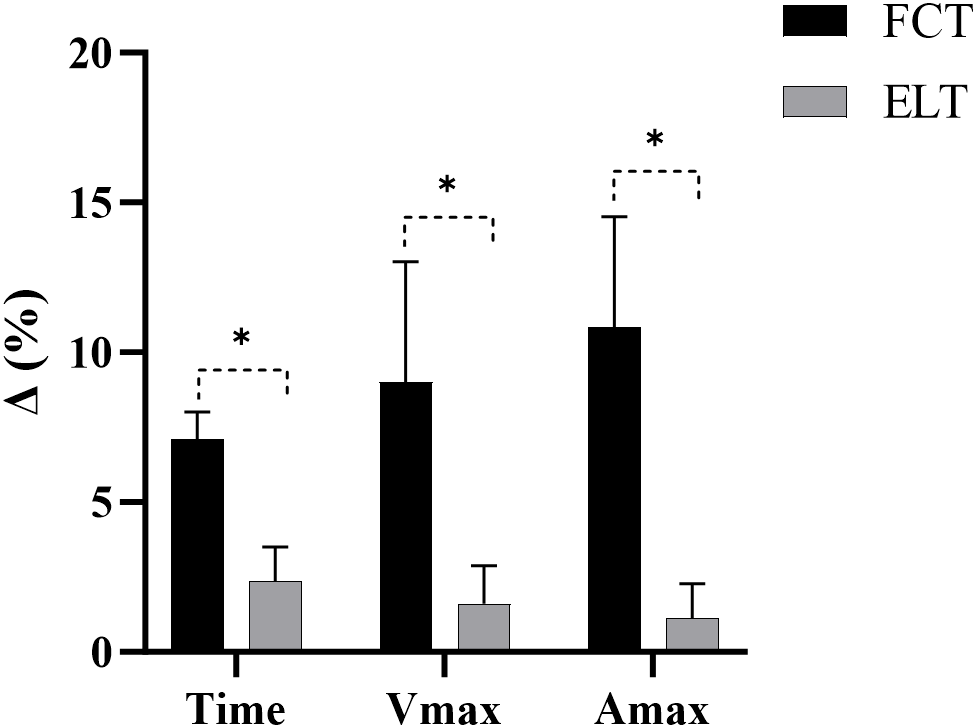
The growth rate of 10-meter sprint.Amax, maximal acceleration; Vmax, maximal velocity; *p<0.05; Δ, growth rate.

The FCT group increased by 7.11% (from 1.94 to 1.80s), compared to 2.35% (from 1.93 to 1.88s) in the ELT group (**Figure 5**).

#### 3.2.2 Vmax

A significant interaction effect between time and group was observed (F = 49.28, p< 0.001, $\eta_{\text{p}}^{2}$ = 0.638, large effect). The main effect of time showed a significant difference (F = 98.04, p< 0.001, $\eta_{\text{p}}^{2}$ = 0.778, large effect). The main effect of the group showed a significant difference (F = 18.95, p< 0.001, $\eta_{\text{p}}^{2}$ = 0.404, large effect) (**Figure 4**).

The FCT group increased by 9.01% (from 7.49 to 8.17m/s), compared to 1.60% (from 7.26 to 7.38m/s) in the ELT group (**Figure 5**).

#### 3.2.3 Amax

A significant interaction effect between time and group was observed (F = 127.31, p< 0.001, $\eta_{\text{p}}^{2}$ = 0.820, large effect). The main effect of time showed a significant difference (F = 189.19, p< 0.001, $\eta_{\text{p}}^{2}$ = 0.871, large effect). The main effect of the group showed a significant difference (F = 7.71, p = 0.010, $\eta_{\text{p}}^{2}$ = 0.216, large effect) (**Figure 4**).

The FCT group increased by 10.84% (from 7.26 to 8.03m/s^2^), compared to 1.12% (from 7.12 to 7.20m/s^2^) in the ELT group (**Figure 5**).

### 3.3 1RM back squat

**Figure 6:**
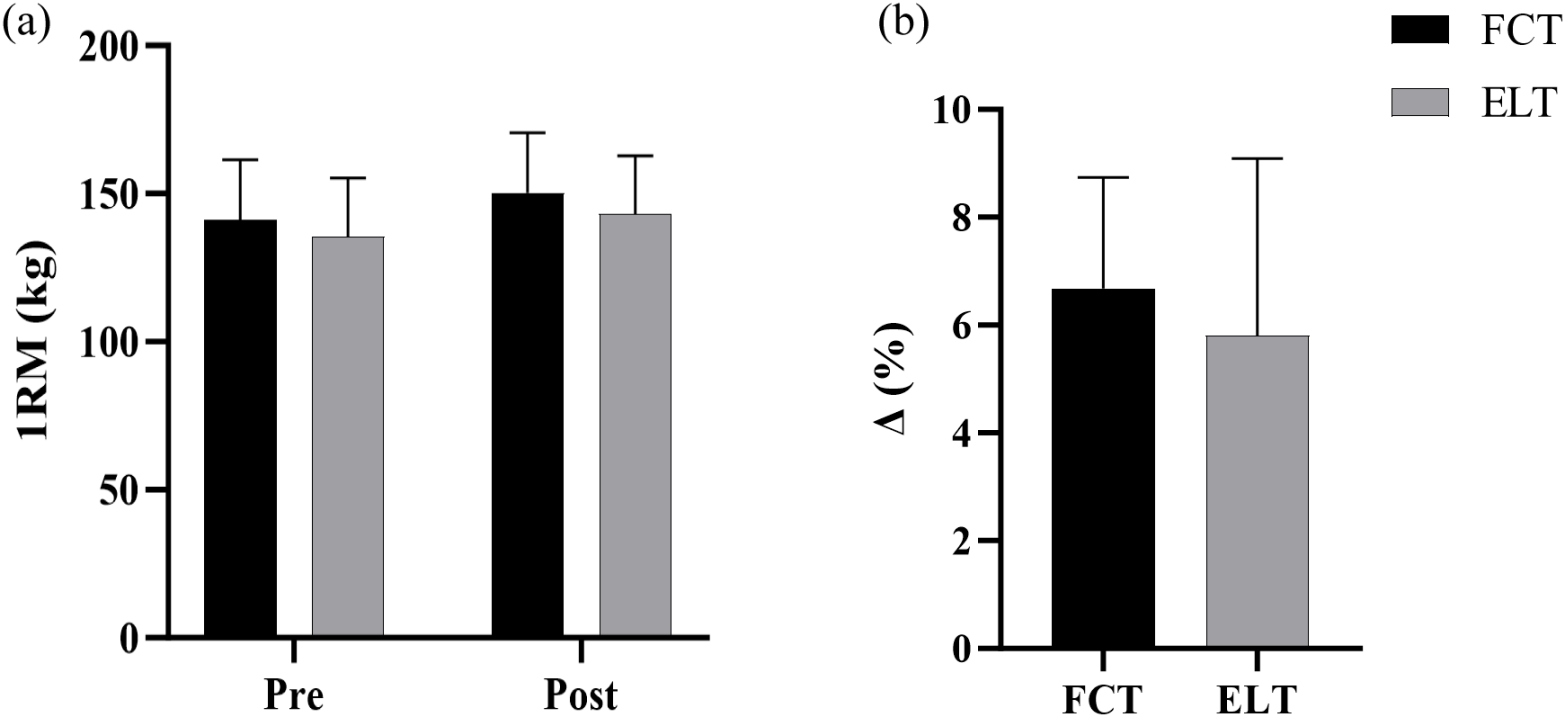
Effect and growth rate of FCT on the 1RM back squat. **(a)** 1RM, **(b)** growth rate. 1RM, one repetition maximal; **Δ**, growth rate.

A non-significant interaction effect between time and group was observed (F = 1.59, p = 0.218, $\eta_{\text{p}}^{2}$ = 0.054, small effect). The main effect of time showed a significant difference (F = 201.40, p< 0.001, $\eta_{\text{p}}^{2}$ = 0.878, large effect). The main effect of the group showed a non-significant difference (F = 0.80, p = 0.379, $\eta_{\text{p}}^{2}$ = 0.028, small effect) (**Figure 6**).

The FCT group increased by 6.67% (from 141.00 to 150.20kg), compared to 5.89% (from 135.33 to 143.03kg) in the ELT group (**Figure 6**).

### 3.4 Load-velocity

#### 3.4.1 20% 1RM

**Figure 7:**
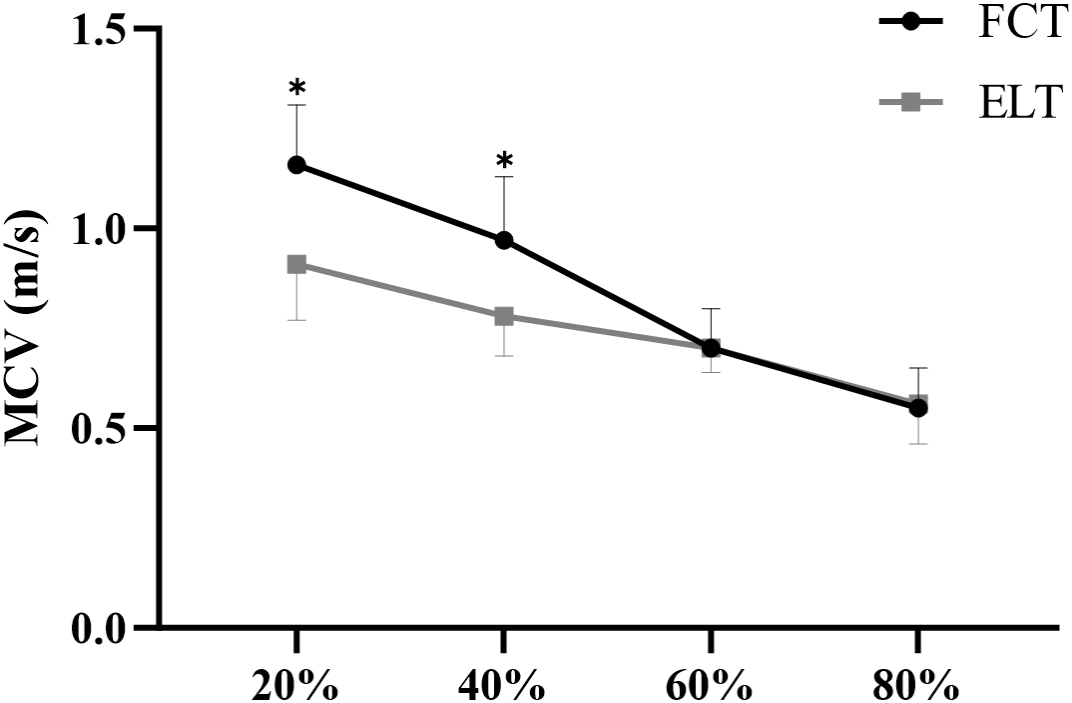
Effect of FCT on the MCV. MCV, mean concentric velocity; *p<0.05.

A significant interaction effect between time and group was observed (F = 44.72, p< 0.001, $\eta_{\text{p}}^{2}$ = 0.615, large effect). The main effect of time showed a significant difference (F = 112.22, p< 0.001, $\eta_{\text{p}}^{2}$ = 0.800, large effect). The main effect of the group showed a significant difference (F = 9.05, p = 0.005, $\eta_{\text{p}}^{2}$ = 0.244, large effect) (**Figure 7**).

**Figure 8:**
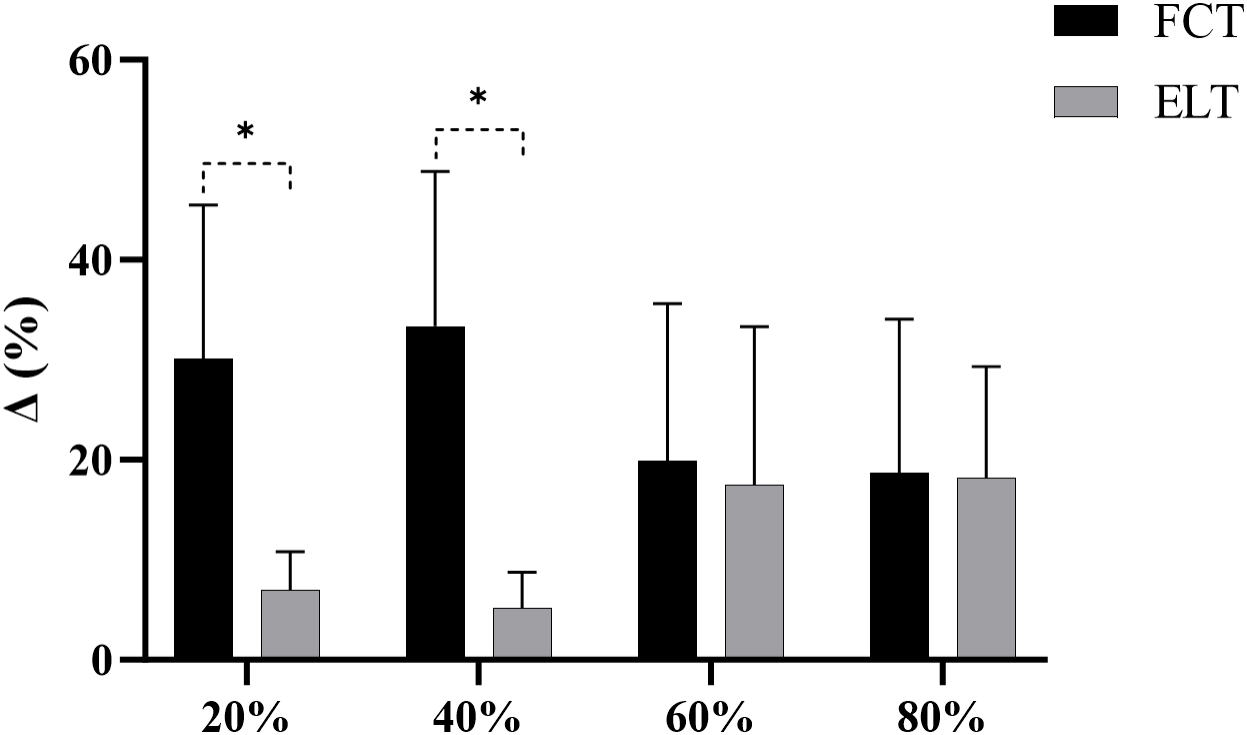
The growth rate of MCV. *p<0.05; Δ, growth rate.

The FCT group increased by 30.12% (from 0.91 to 1.16m/s), compared to 6.99% (from 0.85 to 0.91m/s) in the ELT group (**Figure 8**).

#### 3.4.2 40% 1RM

A significant interaction effect between time and group was observed (F = 64.56, p< 0.001, $\eta_{\text{p}}^{2}$ = 0.697, large effect). The main effect of time showed a significant difference (F = 123.71, p< 0.001, $\eta_{\text{p}}^{2}$ = 0.815, large effect). The main effect of the group showed a significant difference (F = 4.69, p = 0.039, $\eta_{\text{p}}^{2}$ = 0.143, large effect) (**Figure 7**).

The FCT group increased by 33.40% (from 0.73 to 0.97m/s), compared to 5.19% (from 0.74 to 0.78m/s) in the ELT group (**Figure 8**).

#### 3.4.3 60 %1RM

A non-significant interaction effect between time and group was observed (F = 0.32, p = 0.579, $\eta_{\text{p}}^{2}$ = 0.011, small effect). The main effect of time showed a significant difference (F = 56.10, p< 0.001, $\eta_{\text{p}}^{2}$ = 0.667, large effect). The main effect of the group showed a non-significant difference (F = 0.20, p = 0.655, $\eta_{\text{p}}^{2}$ = 0.007, trivial effect) (**Figure 7**).

The FCT group increased by 19.86% (from 0.59 to 0.70m/s), compared to 17.54% (from 0.61 to 0.70m/s) in the ELT group (**Figure 8**).

#### 3.4.4 80 %1RM

A non-significant interaction effect between time and group was observed (F = 0.02, p = 0.900, $\eta_{\text{p}}^{2}$ = 0.001, trivial effect). The main effect of time showed a significant difference (F = 58.19, p< 0.001, $\eta_{\text{p}}^{2}$ = 0.675, large effect). The main effect of the group showed a non-significant difference (F = 0.11, p = 0.738, $\eta_{\text{p}}^{2}$ = 0.004, trivial effect) (**Figure 7**).

The FCT group increased by 18.72% (from 0.46 to 0.55m/s), compared to 18.24% (from 0.47 to 0.56m/s) in the ELT group (**Figure 8**, **Table 2**).

**Table 2:** The effects of the FCT group and ELT group on the athletic performance of participants.

Variable	Group	Pre	Post	Δ% (mean)	*p*			Bonferroni *p*
					Time	Group	Interaction	FCT vs ELT at post
Vertical jump	CMJ (cm)							
	FCT	44.62 ± 5.12	49.84 ± 4.48	12.11	*<0.001*	*0.035*	*<0.001*	*0.002*
	ELT	42.75 ± 5.20	43.72 ± 5.23	2.31				
	SJ (cm)							
	FCT	42.47 ± 4.22	49.04 ± 4.20	15.68	*<0.001*	*0.041*	*<0.001*	*0.003*
	ELT	40.40 ± 7.10	42.03 ± 7.08	4.16				
10-meter sprint	Time (s)							
	FCT	1.94 ± 0.05	1.80 ± 0.05	7.11	*<0.001*	*0.032*	*<0.001*	*<0.001*
	ELT	1.93 ± 0.05	1.88 ± 0.05	2.35				
	Vmax (m/s)							
	FCT	7.49 ± 0.25	8.17 ± 0.35	9.01	*<0.001*	*<0.001*	*<0.001*	*<0.001*
	ELT	7.26 ± 0.38	7.38 ± 0.35	1.60				
	Amax (m/s^2^)							
	FCT	7.26 ± 0.53	8.03 ± 0.51	10.84	*<0.001*	*0.010*	*<0.001*	*<0.001*
	ELT	7.12 ± 0.47	7.20 ± 0.43	1.12				
1RM squat	FCT	141.00 ± 20.44	150.20 ± 20.36	6.67	*<0.001*	*0.379*	*0.218*	*0.328*
	ELT	135.33 ± 19.11	143.03 ± 19.04	5.89				
MCV	20% 1RM (m/s)							
	FCT	0.91 ± 0.16	1.16 ± 0.15	30.12	*<0.001*	*0.005*	*<0.001*	*<0.001*
	ELT	0.85 ± 0.13	0.91 ± 0.13	6.99				
	40% 1RM (m/s)							
	FCT	0.73 ± 0.13	0.97 ± 0.16	33.40	*<0.001*	*0.039*	*<0.001*	*<0.001*
	ELT	0.74 ± 0.10	0.78 ± 0.10	5.19				
	60% 1RM (m/s)							
	FCT	0.59 ± 0.08	0.70 ± 0.10	19.86	*<0.001*	*0.655*	*0.579*	*0.892*
	ELT	0.61 ± 0.08	0.70 ± 0.06	17.54				
	80% 1RM (m/s)							
	FCT	0.46 ± 0.05	0.55 ± 0.10	18.72	*<0.001*	*0.738*	*0.900*	*0.827*
	ELT	0.47 ± 0.07	0.56 ± 0.09	18.24				

**CMJ**, countermovement jump; **MCV**, mean concentric velocity; **SJ**, squat jump; **1RM**, one repetition maximal.

## 4 Discussion

This is the first study to investigate the effects of FCT on lower-limb athletic performance in male college badminton players. The main findings showed that, compared to ELT, FCT effectively improved vertical jump, 10-meter sprint, and MCV from 20 to 40% of 1RM, but not superior to enhance 1RM back squat or MCV from 60 to 80% of 1RM in male college badminton players. These findings suggested that FCT enhances lower-limb explosive power, acceleration, and rapid strength at low loads, but it does not further optimize maximum strength.

This also aligns with our earlier observations, which demonstrated that FCT is beneficial for enhancing lower-limb explosive power in martial athletes (Chen et al., 2025). Elbadry et al. demonstrated that an eight-week FCT program significantly enhanced CMJ performance among female college athletes (Elbadry et al., 2019). Furthermore, Turkarslan et al. showed that a six-week FCT program significantly improved 30-meter sprint times for male soccer players (Türkarslan and Deliceoglu, 2024). The observed increase in lower-limb explosive power could be attributed to the FCT providing comprehensive neuromuscular stimulation across the entire range of the F-V curve (Cormier et al., 2022). Heavy compound exercises could serve as a high-threshold primer by increasing corticospinal excitability, recruiting high-threshold motor units, enhancing light-chain phosphorylation, and raising muscle temperature, creating a short window for PAPE (Bishop, 2003; Davies et al., 2015; Blazevich and Babault, 2019; Zimmermann et al., 2020; Gordon et al., 2024). These mechanical adaptations enable eccentric energy to be more fully converted into concentric impulse, directly increasing take-off speed (Hirayama et al., 2017; Kubo et al., 2021). The light-to-moderate load exercises (20 to 40% of 1RM) shift the F-V curve to the right, increase the rate of force development (RFD) within 0–100ms, and decrease tendon slack time (Tsoukos et al., 2018; Kim et al., 2021). These effects facilitate a better transfer of contractile shortening into movement speed, from the hip to the ankle (Arnold et al., 2013; Van Hooren and Bosch, 2016; Kubo et al., 2021). The assisted plyometric exercises expose the neuromuscular system to higher speeds, improving feed-forward timing and reducing co-contraction during brief propulsion events (Tufano and Amonette, 2018; Peng et al., 2023; Koźlenia and Domaradzki, 2024). Collectively, these underlying mechanisms enhance maximum velocity, resulting in faster and more efficient unassisted takeoffs (Aagaard et al., 2002; Argus et al., 2011; Maffiuletti et al., 2016; Sanpasitt and Apanukul, 2023; Behm et al., 2024). Notably, improvements in low-load MCV (20 to 40%1RM), early acceleration metrics (10-meter time, V_max_, A_max_), and vertical jump (CMJ, SJ) also support these underlying mechanisms. Additionally, FCT may promote efficient excitation-contraction coupling in skeletal muscle and tendon stiffness (Lichtwark and Wilson, 2006; Raiteri et al., 2018; Holzer et al., 2024); studies showed that the enhanced intermuscular coordination from complex training reduces synergistic activation of antagonist muscles (Heald et al., 2018; Santos et al., 2021; Ramirez-Campillo et al., 2023). These potential benefits may enhance acceleration and impulse during concentric contractions, thereby improving jump height and acceleration.

Contrary to expectations, this study did not find a significant difference between groups in 1RM and MCV from 60 to 80% 1RM. This outcome is contrary to that of Rebelo et al. who found that a six-week FCT program significantly enhanced 1RM squat but did not significantly improve MCV velocity from 60 to 80% 1RM compared to conventional complex training in young female artistic roller-skating athletes (Rebelo et al., 2023). A possible explanation for this might be maximal strength benefits from regular exposure to high intensities (>85% 1RM) combined with adequate set volume and increased time under tension (TUT) (Burd et al., 2012; Bernárdez-Vázquez et al., 2022). The control group in this study employed an ELT design to ensure consistency in the high-intensity exercise regimen throughout the eight-week intervention. These findings align with the concept that neuromuscular adaptation is specific to both the dose and the task (Rhea et al., 2003; Škarabot et al., 2021). Schoenfeld et al. reported that lifting at intensities above 85% 1RM for multiple sets is crucial for optimizing muscle strength adaptations (Schoenfeld et al., 2021). The FCT protocol focused on movement velocity and neural activation rather than sustained load, resulting in reduced overall mechanical strain. Additionally, high-frequency plyometric training in the FCT may lead to neuromuscular fatigue, reducing motor unit recruitment efficiency and impairing the expression of maximum strength (Hung et al., 2025).

In summary, these findings suggest that FCT effectively enhances vertical jump and early acceleration rather than maximum strength. The findings have important implications for sport-specific performance, especially in badminton, where athletes need to generate propulsive velocity and impulse. Practically, coaches can use FCT to improve vertical jump and early acceleration, scheduling two weekly sessions with at least 48 hours of recovery.

### 4.1 Limitations

This study has limitations, including a small sample of male college badminton players, which limits its applicability to females. No long-term follow-up. The lack of physiological and neuromuscular biomarker assessments also restricts insights into training mechanisms. Future research should consider gender differences and use biomarkers to evaluate FCT effects on badminton players.

## 5 Conclusion

This study provides evidence that French Contrast Training (FCT) may be more effective than equal-load training (ELT) for enhancing vertical jump and early acceleration, but it was not superior to ELT for maximal strength development among male college badminton players. These findings suggest a potential role for FCT in periodized training programs. The sample size was limited to male college badminton athletes, so the findings should be interpreted cautiously. Future research should confirm these conclusions across various genders and training levels and investigate the long-term effects of FCT using biomechanical and physiological indicators.

## Data Availability

The original contributions presented in the study are publicly available. This data can be found here: Science Data Bank (Science DB) https://doi.org/10.57760/sciencedb.28819.
