# Supplementary material for: Effects of an eight-week French contrast training program on lower-limb explosive power, acceleration, and muscle strength in male college badminton players
Source: Front Physiol. 2026 Apr 2;17:1777883. doi: 10.3389/fphys.2026.1777883 (PMC13084729; doi:10.3389/fphys.2026.1777883)
Supplement: Supplementary file 1 [file Table1.docx]

Supplementary Material

**Effects of an Eight-Week French Contrast Training Program on** **Lower-Limb** **Explosive Power,** **Acceleration, and Muscle Strength in** **Male College Badminton Players**

Kaixiang Zhou^1，†^, Ruting Lin^2, †^, Ziren Zhao^1^^, †^, Na Yu^1^, Xin Zheng^1^, Jinhong Li^1^, Nijiao Deng^1^, Yu'an Sun^3，*^

^1^ College of Physical Education and Health Science, Chongqing Normal University, Chongqing, China;

^2^ College of Education, Beijing Sport University, Beijing, China;

^3^ College of Sports and Health, Chengdu University of Traditional Chinese Medicine, Chengdu, Sichuan, China;

*** Correspondence:**

Yu'an Sun, College of Sports and Health, Chengdu University of Traditional Chinese Medicine, Chengdu, Sichuan, China; e-mail address: ssssyyyyaaaa@163.com

**^†^ Equal contribution and first authorship:** These authors contributed equally to this work and share first authorship

**1 Supplementary Tables**

1.1 Table S1 Within-session reliability of performance measures across three repeated trials.

Table S1 Within-session reliability of performance measures across three repeated trials.

| Outcome | Session | ICC (3,3) | 95%CI |
| --- | --- | --- | --- |
| Vmax (m/s) | Pre | 0.910 | 0.833, 0.955 |
| Vmax (m/s) | Post | 0.955 | 0.916, 0.977 |
| Amax (m/s²) | Pre | 0.952 | 0.910, 0.976 |
| Amax (m/s²) | Post | 0.965 | 0.936, 0.983 |
| 10-m time (s) | Pre | 0.843 | 0.713, 0.916 |
| 10-m time (s) | Post | 0.848 | 0.719, 0.924 |
| SJ height (cm) | Pre | 0.988 | 0.978, 0.994 |
| SJ height (cm) | Post | 0.989 | 0.979, 0.994 |
| CMJ height (cm) | Pre | 0.983 | 0.968, 0.991 |
| CMJ height (cm) | Post | 0.983 | 0.969, 0.992 |
